# Left ventricular dysfunction postsurgical patent ductus arteriosus ligation in children: predictor factors analysis

**DOI:** 10.1186/s13019-019-0990-z

**Published:** 2019-09-18

**Authors:** Mohamed Abdel-Bary, Khaled Abdalla Abdel-Baseer, Ahmed Fathy Abdel-Latif, Mohamed Abdella Abdel-Naser, Mahmoud Nafie, Karam Mosallam Eisa

**Affiliations:** 1 Department of cardiothoracic surgery, Qena Faculty of Medicine, South Valley University, Safaga Road, Qena, 83523 Egypt; 2 Department of Pediatrics, Qena Faculty of Medicine, South Valley University, Qena, Egypt; 3 Department of Anaesthesia and ICU, Qena Faculty of Medicine, South Valley University, Qena, Egypt; 4 Department of Anaesthesia and ICU, Faculty of Medicine, Assiut University, Asyut, Egypt; 5 National heart institute, Cairo, Egypt

**Keywords:** PDA ligation, Congenital heart disease, LV systolic function and dimensions, LA/Ao ratio

## Abstract

**Objective:**

To identify the predictor factors of left ventricular (LV) dysfunction following patent ductus arteriosus (PDA) surgical ligation.

**Background:**

PDA is viewed as a noticeable amongst the most widely recognized congenital heart defects in children and its closure is responsible for many hemodynamic changes that require intervention and care.

**Methods:**

A retrospective study included fifty children with isolated PDA treated by surgical ligation from June 2015 to June 2018. The LV dimensions and systolic function were assessed by two-dimensional echocardiography pre and post PDA ligation. All cases were followed-up on the first-day, 1 month and 6 months post ligation.

**Results:**

The mean age of cases was 15.78 ± 7.58 months and 72% were females. The mean duct size was 4.08 ± 1.25 mm. There was a marked decrease in LVEDd, LA/Ao, EF and FS in the first-day post ligation contrasted with pre ligation values. Moreover, an amazing decline in LVEDd and LA/Ao ratio was observed 1 month post ligation contrasted with the early post ligation status with asynchronous improvement of FS and EF at one and 6 months postoperatively.

**Conclusion:**

PDA ligation is associated with a noteworthy LV systolic dysfunction within the first day post ligation; that in a significant number of patients may require anti-failure measures, prolong the hospital stay and necessitate a regular follow up and monitoring of LV function. PDA size, age, preoperative LVEDd and FS can be considered as predictor factors for suspicion of acute decrease in the LV systolic function early post PDA ligation.

**Trial registration:**

ClinTrial.Gov NCT04018079.

## Background

Ductus arteriosus is a shunt in the fetal circulation between the pulmonary artery and the proximal descending aorta [1]. A confined PDA is a noticeable amongst the most common congenital heart defects (CHD); as its incidence up to 8 for every 10,000 live births among term infants [2, 3]. The left to right shunt via a hemodynamically noteworthy PDA causes pulmonary over-flooding that result in the left ventricle (LV) volume over-burden and remodeling, and it compensates by expanding stroke volume. Congestive heart failure (CHF) occurs in cases with greater shunts [4].

PDA may be associated with cardiac or respiratory morbidity. So, the closure should be performed once diagnosed. Surgical ligation indicated in huge PDA and not appropriate for percutaneous intervention as well as in developing countries [5–7].

Post PDA closure syndrome is the cardiorespiratory instability that may occur within the first 12 h post closure. It happens due to LV dysfunction and vascular tone dysregulation. Its occurrence and indicators have been estimated in recent studies. They reported a decrease in fraction shortening (FS) and ejection fraction (EF) associated with an increase in systemic vascular resistance and a sudden reduction in preload [8–11].

However, the data about the preoperative factors that can anticipate this syndrome in children is scanty. The search for noninvasive preoperative predictors of LV dysfunction post PDA ligation is needed. The current study evaluated the pre and postoperative echocardiographic changes to identify the preoperative predictor factors of LV dysfunction following PDA surgical ligation in children.

## Methods

### Study population

This retrospective observational cohort study investigated fifty children who underwent isolated PDA surgical ligation at cardiothoracic surgery department, Qena University Hospital, Egypt between June 2015 to June 2018. The follow up was done by paediatricians at cardiac surgery at the same hospital. Their mean age at intervention was 15.78 ± 7.58 months, body surface area (BSA) 0.43 ± 0.03 and 36 (72%) were females. They were referred for surgical closure from pediatricians late; because they presented late in these rural areas of Upper Egypt. The indication for surgical ligation at our center was cases who had clinical as well as echocardiographic proof of hemodynamically critical isolated PDA (large shunt volume PDA, Qp /Qs > 2, LV dilatation, diastolic pressure gradient< 25 mmHg). The exclusion criteria were the following: preterm babies; patients with other congenital heart defects; patients with silent PDA and patients with irreversible pulmonary vascular disease. The mean duct diameter was 4.08 ± 1.25 mm. All of our cases were hemodynamically stable and all of them were extubated on table. There was no need for mechanical ventilation. All cases completed the follow up. Also, we didn’t report any mortality. The study conforms to the ethical standards of the Helsinki Declaration and approval was obtained from the institutional ethics committee of Qena Faculty of Medicine.

### Surgical technique

PDA ligation was performed under general anaesthesia after pre-operative anaesthetic evaluation. After single endotracheal tube anaesthesia induction; children were placed on the right lateral recumbent position fixed with adhesive plaster and a pad under the chest, the left arm raised above the head. A left mini-thoracotomy incision is done parallel to the medial border of the scapula and entrance to the thoracic cavity was via the third or fourth intercostal space. Cautiously, the ductus is identified and dissected carefully. Then it was doubly ligated with silk ligature (2/0 or 0). An intercostal tube inserted during operation and removed within 48 h if no drainage presents.

### Echocardiography

Transthoracic 2D echocardiography was done with the child in the recumbent position pre ligation; at 1 day, 1 month and 6 months post ligation. Some cases required sedation during the examination. They didn’t receive any inotropes during examination. Each echocardiography was done by two independent operators. The five cardiac cycles were acquired and digitally stored for analysis. The LV end-diastolic dimension (LVEDd), LV end-systolic dimension (LVESd) and left atrial to aortic diameter ratio (LA/Ao) were measured in the parasternal long-axis view and indexed to the body surface area (BSA). Fractional shortening (FS) was calculated as {(LVEDd-LVESd)/LVEDd} × 100. PDA diameter was measured using color-Doppler in short axis parasternal view and modified high parasternal views, measuring the narrowest point. On studying the predictors for the occurrence of postligation LV dysfunction, we classified our patients into two gatherings as indicated by the postoperative FS % based on a definition of LV systolic dysfunction; group I with FS ≤ 29% and group II with FS > 29% [12].

### Descriptive statistical analysis

Data were arranged and analyzed utilizing Version 20 of the SPSS program (Statistical Package for Social Sciences). Continuous variables were compared using the Student paired t-test and are expressed as mean values ± standard deviation. Pearson Chi-Square tests were used to detect differences among groups for categorical variables. The relationship between PDA size and changes in echocardiographic parameters was verified using the Pearson linear correlation and the linear regression analysis. *P*-value of < 0.05 was considered of significance.

## Results

PDA was ligated successfully with no residual flow in all cases. In group I, nine cases (64%) suffered from CHF and dyspnea; they were treated with oral antifailure medications (diuretics, angiotensin-converting-enzyme inhibitors (ACEIs) and/or digoxin). They were completely improved within 2-3 weeks. During follow up, no latent arrhythmias were detected. The respiratory tract infection and the postoperative hospital stay were recorded (Table 3). Group I cases had more hospital stays as some of them developed dyspnea and post-operative CHF; and large sized PDA cases were more in this group. So, they were more susceptible to increased pulmonary flow and chest infection. There were no deaths, bleeding or any other life-threatening complications.

In the first-day post PDA ligation, there was a significant decrease in LVEDd, LA/Ao, EF and FS contrasted with the pre ligation values. However, 1 month post ligation a significant decrease in LVEDd, LVESd and LA/AO ratio was found contrasted with the pre ligation values, while EF and FS was unchanged (Table 1).

**Table 1:** Comparison between the pre & post ligation LV systolic function, dimensions & LA/AO ratio

Parameters	Pre ligation (I) (*n* = 50)	First-day post ligation (II) (*n* = 50)	One-month post ligation (III) (*n* = 50)	Six-months post ligation (IV) (*n* = 50)	*P*-value I & II	*P*-value I & III	*P*-value II &III	*P*-value II &IV	*P*-value III &IV
LVEDd (mm)	3.25 ± 0.35	2.98 ± 0.25	2.93 ± 0.26	2.90 ± 0.22	< 0.001	< 0.001	< 0.05	< 0.001	< 0.05
LVESd (mm)	2.02 ± 0.30	1.97 ± 0.23	1.91 ± 0.18	1.87 ± 0.21	NS	< 0.05	< 0.05	< 0.001	< 0.05
LA/AO ratio	1.67 ± 0.32	1.39 ± 0.28	1.29 ± 0.22	1.25 ± 0.21	< 0.001	< 0.001	< 0.001	< 0.001	< 0.05
FS (%)	36.73 ± 3.09	32.40 ± 3.61	37.4 ± 2.79	37.92 ± 2.83	< 0.01	NS	< 0.01	< 0.001	NS
EF (%)	61.12 ± 3.82	58.18 ± 3.46	60.78 ± 2.95	61.02 ± 2.25	< 0.001	NS	< 0.001	< 0.001	NS

The LV systolic function and dimensions were improved markedly at 1 month and 6 months post ligation follow up contrasted with pre ligation measures. Furthermore, the same improvement was noticed at 1 month follow up contrasted with the first-day post ligation values. Also, the same findings at 6 months contrasted with one-month post ligation parameters (Table 1).

As our study distinguished noteworthy decrease in LVEDd in early post PDA ligation contrasted with pre-ligation diameters, a linear regression analysis was done to clarify the impact of PDA size on the LV systolic function and LVEDd changes from pre ligation to early post ligation (Table 2).

**Table 2:** Effect of PDA size on changes in LV function and LVED dimensions

Parameter	LVEDd change		FS change		EF change	
PDA size (mm)	R	*P* value	R	*P* value	R	*P* value
	-0.703	< 0.001	-0.645	< 0.01	-0.742	< 0.001

*R* linear regression

The predictor factors that may identify the LV dysfunction post ligation were postulated in Table 3. Cases with FS ≤ 29% indicated essentially higher age, larger ductus size and bigger LVEDd and a lower ratio of FS and EF than others. Likewise, a significantly higher ratio of recurrent chest infections and prolonged hospital stay were found in cases with FS ≤ 29% than others.

**Table 3:** Preoperative predictors of LV systolic dysfunction post PDA ligation

Parameter	Cases with FS ≤ 29% (*n* = 14) Group I	Cases with FS > 29% (*n* = 36) Group II	*P* value
Age (months)	26.50 ± 4.41	17.58 ± 4.89	< 0.001
PDA size (mm)	5.42 ± 0.75	3.55 ± 0.99	< 0.001
BSA	0.44 ± 0.04	0.43 ± 0.33	NS
Sex (male /female ratio)	4/10	10/26	NS
LVEDd (mm)	3.57 ± 0.21	3.13 ± 0.33	< 0.001
FS (%)	34.96 ± 3.49	37.41 ± 2.67	< 0.05
EF (%)	59.50 ± 4.23	61.75 ± 3.50	NS
Hospital stay (days)	6.68 ± 0.70	4.04 ± 0.54	< 0.001
Recurrent chest infections (%)	57	25	< 0.05
CHF	9 (64%)	0	< 0.001

*BSA* Body surface area

*LVEDd* Left ventricular end diastolic dimension

*FS* Fractional shortening

*EF* Ejection fraction

*CHF* Congestive heart failure

**Fig. 1:**
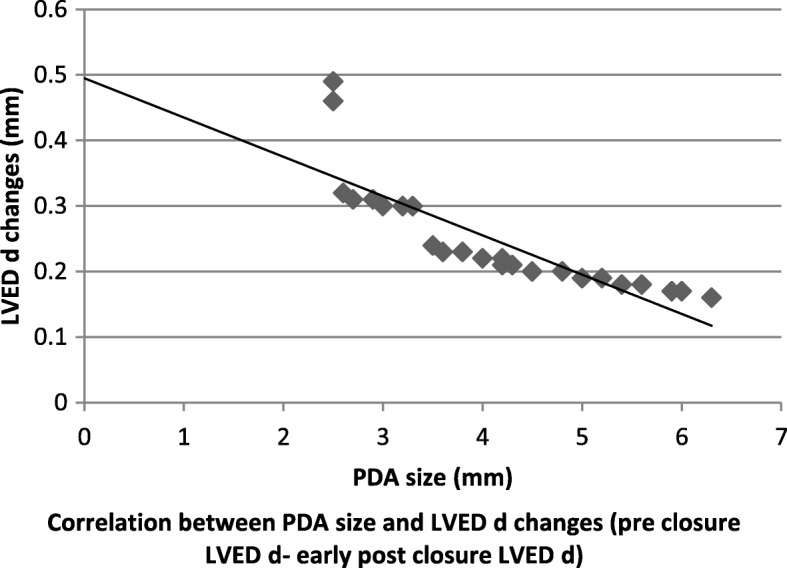
correlation between PDA size and LVED d changes (pre closure LVED d – early post closure LVED d)

**Fig. 2:**
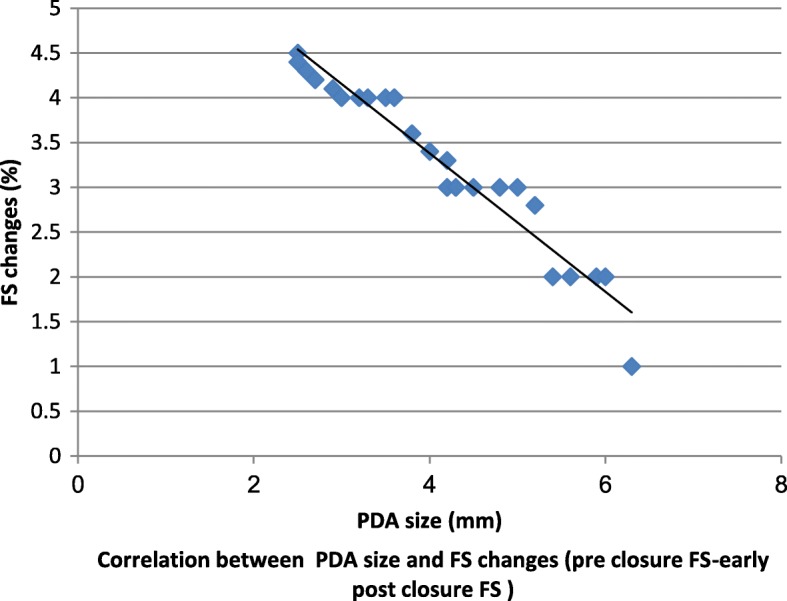
correlation between PDA size and FS changes (pre closure FS – early post closure FS)

Furthermore, a negative correlation was identified between the ductus size and pre and post ligation changes in LVEDd and FS (Figs. 1 and 2).

## Discussion

Our results of early post-PDA ligation, there was a significant decrease in LVEDd and LV systolic function which improved remarkably after 1 month returning to approximate pre ligation measures. This was in concurrence with the outcome of other studies [13–15]. Based on the Frank-Starling’s Law, the increased preload causes increased contractility by stretching of LV muscle fibers to overcome significant left-to-right shunt and maintain systemic circulation and therefore increased systolic function. PDA ligation results in an abrupt decrease in LV preload and hence reducing stretching of muscle fiber decreasing FS a marker of systolic dysfunction. Another hypothesis explaining LV systolic dysfunction is that ductus ligation causes increased afterload by the low-resistance pulmonary circulation from LV outflow circulation. This synchronous decrease in the LV preload and increment in the afterload may prompt LV systolic dysfunction.

Galal et al. [14], demonstrated that children with PDA had significant deterioration in FS immediately after PDA closure, which was followed by normalization within 6 months. They added that children with acute disturbance of LV function following large PDA closure may experience symptoms of exercise intolerance and necessitate therapy with afterload-reducing drugs like ACEIs. Contrasted with the pre ligation ductus parameters in this research, a significant reduction in the LVEDd was noted; while there was no significant distinction in the LVESd immediately post ligation. However, an additional improvement in the LV dimensions was detected during follow-up. The early reduction in the LVEDd and a late reduction in the LVESd resulted in a change in the FS when pre and post-ligation follow-up values were compared. This was in accordance with previous studies [15, 16]. Elsheikh et al. [17], detected that most PDA patients showed a significant decrease in LV systolic function and dimensions post closure in comparison to pre-closure. They hypothesized that prior to closure, the LV pumps blood both into the high-resistance systemic circulation and through PDA into the low-resistance pulmonary circulation, while post PDA closure it ejects blood into the high-resistance systemic circulation only. Therefore, they conclude that an increase in the LV afterload post-closure,may be the contributing factor of the reduced systolic function.

Our study detected that spontaneous resolution of LV systolic dysfunction manifested by improvement of FS and EF as pre ligation state starts within 1 month with more improvement within 6 months which is consistent with prior studies [14, 18]. In contrary to our study, Jeong et al. [6], detected a persistent decrease in FS and EF in 11.1% of adult patients for whom PDA closure was done; this discrepancy might be clarified by the difference of age groups between both studies. Additionally, in another study done by Hussain et al. [19], all patients developed dysfunction immediately after device closure, 87.8% patients recovered within 3 months and 92.7% within 6 months and only 7.3% had mild dysfunction beyond 6 months. They hypothesized that LV volume overload for longer duration may induce irreversible structural changes in the LV myocardium that impair recovery even after overloading condition terminated.

Few studies were done to evaluate predictors for the occurrence of LV dysfunction following PDA surgical closure. In our study, in spite that all patients showed a decrease in FS than preoperative values, we noticed that the group that suffered from more deterioration in LV function had a larger PDA size than others. Recently, Kiran et al. [20], found that the PDA size affects the degree of LV function, they classify their patients into four categories as indicated by the measurement of PDA size. They found normal LV function at pre-closure baseline but following closure detected that the group of the largest PDA size was the most suffering from LV dysfunction with a significant correlation. Also, we detected more LV dysfunction in those with older age than others reflecting the fact that delayed surgical closure leads to the more prolonged left to right shunt hence, more LV overload and dysfunction especially with increased LV afterload induced by PDA ligation.

Also, significantly preoperative low values of LVEDds were detected in those with postoperative FS ≤ 29% (group I). This observation was consistent with Kim et al. [16] and Agha et al. [21] Also Hussain et al. [19], study detected a higher LVEDd in low EF patients than others and this was well correlated with post procedure LV dysfunction and added that LVEDd decreased on follow up as LV systolic function improved. Additionally, we found a significantly lower ratio of pre ligation FS in group I and we think that this finding is a reasonable reflection of higher LVEDd in this group according to FS calculation equation. Group, I cases showed a significantly higher rate of recurrent chest infections more than group II. This may reflect the impact of a larger PDA size in this group on the pulmonary circulation. It is notable that if the PDA stays open for long periods, it maintains an increased pulmonary blood flow, capillary pressure, and lung fluid enhancing the hazard for lung disease development [22]. We noticed a negative correlation between the ductus size and LVEDd, FS and EF changes. Because in the early post ligation period, we identified that the bigger PDA size, the more decrease in LVEDd, FS and EF. These findings were steady with perceptions made by Amoogzar et al. [23], and El sheddony et al. [15]

The most important limitation of our study is the lack of a control group. Therefore, although serial echocardiograms were performed before and after surgical PDA ligation and each patient behave as his or her own control, we could not control for the impact of anesthesia or the stress of surgery on cardiac function. Other limitations of our study include the retrospective nature of the study design that might not be the proper method for confirmation of studied relationships and the relatively small number of the studied groups.

## Conclusions

PDA ligation is associated with a significant reduction of LV systolic function in the first-day post ligation; that in a significant number of patients may require anti-failure measures, prolong the hospital stay and necessitate a regular follow up and monitoring of LV function. PDA size, age, preoperative LVEDd and FS can be considered as predictor factors for suspicion of acute decrease in the LV systolic function early post PDA ligation.

## Data Availability

The datasets used or analyzed during the current study are available from the corresponding author on reasonable request.

## Abbreviations

ACEIs: Angiotensin-converting-enzyme inhibitors
BSA: Body surface area
CHD: Congenital heart defects
CHF: Congestive heart failure
EF: Ejection fraction
FS: Fractional shortening
LA/Ao: Left atrial to aortic diameter ratio
LV: Left ventricle
LVEDd: Left ventricular end diastolic dimension
LVESd: LV end-systolic dimension
PDA: Patent ductus arteriosus
